# A brief history of bacterial growth physiology

**DOI:** 10.3389/fmicb.2015.00289

**Published:** 2015-04-21

**Authors:** Moselio Schaechter

**Affiliations:** Biology Department, San Diego State University, and Division of Biological Sciences, University of California, San DiegoSan Diego, CA, USA

**Keywords:** growth physiology, Copenhagen school, balanced growth, bacterial physiology, bacterial growth and physiology

## Abstract

Arguably, microbial physiology started when Leeuwenhoek became fascinated by observing a Vorticella beating its cilia, my point being that almost any observation of microbes has a physiological component. With the advent of modern microbiology in the mid-19th century, the field became recognizably distinctive with such discoveries as anaerobiosis, fermentation as a biological phenomenon, and the nutritional requirements of microbes. Soon came the discoveries of Winogradsky and his followers of the chemical changes in the environment that result from microbial activities. Later, during the first half of the 20th century, microbial physiology became the basis for much of the elucidation of central metabolism. Bacterial physiology then became a handmaiden of molecular biology and was greatly influenced by the discovery of cellular regulatory mechanisms. Microbial growth, which had come of age with the early work of Hershey, Monod, and others, was later pursued by studies on a whole cell level by what became known as the “Copenhagen School.” During this time, the exploration of physiological activities became coupled to modern inquiries into the structure of the bacterial cell. Recent years have seen the development of a further phase in microbial physiology, one seeking a deeper quantitative understanding of phenomena on a whole cell level. This pursuit is exemplified by the emergence of systems biology, which is made possible by the development of technologies that permit the gathering of information in huge amounts. As has been true through history, the research into microbial physiology continues to be guided by the development of new methods of analysis. Some of these developments may well afford the possibility of making stunning breakthroughs.

## Ancient history

Arguably, the science of microbial physiology began when Leeuwenhoek first became fascinated by the sight of *Vorticella* beating its cilia. I propose that like most observations of microbes, from the simplest to the most sophisticated, this one has a physiological component. With the advent of modern microbiology in the mid-19th century, this new field of inquiry became conspicuous and recognizable with discoveries of processes such as anaerobiosis and sporulation, along with the recognition of fermentation as a microbiological phenomenon. Soon thereafter came the studies of Winogradsky and his followers on the chemical changes in the environment that result from microbial activities. Later still, during the first half of the 20th century, microbial physiology was a major contributor to biochemistry and played a key role in the elucidation of central metabolism.

The understanding of the physiology of *bacterial growth*, however, lagged behind. Even in the early 1950s, a student of microbiology, like myself, who wished to understand what happens when bacteria grow, was hard put to find useful guideposts. In the textbooks of the day, the focus was on the growth curve, with its depressingly unintelligible sequence of phases and the implication that they represented stages of an obligatory life cycle. Yet, even from the earliest days of microbiology, there were beacons of lucid thinking on the subject. One of Pasteur's first students, Raulin (1869), carried out quantitative growth experiments with the mold *Aspergillus niger* that revealed, surprisingly, its ability to grow on a simple sugar and a few mineral salts. Raulin's minimal medium is not very different from those used today. Pasteur himself believed almost obsessively that the morphology and activities of microbes are conditioned by their environment.

In time, a vast literature on growth experiments accumulated, some fanciful, others exact in intent and meticulous in execution. Notable for its clarity of thought is Henrici's classic (Henrici, 1928) report on how bacteria change in size throughout their growth cycle. Despite such examples of astute insight, a fog continued to envelop growth physiology, fueled by quirky notions. For example, some thought that the yield of bacterial cultures was limited by an entity called “biological space.” Others saw the growth curve as inexorably S-shaped, thus determined by the logistic equation first published by Pierre Verhulst (1845). (I have run into people who believe this to this day.) Throughout this period, the sanctity of the growth curve prevailed. In a 1949 review on growth, even Van Niel (1949) stated: “Nearly all that it is known about the kinetics of growth of microorganisms has been learned from studies of so-called growth curves.”

## Recent history

**Figure 1:**
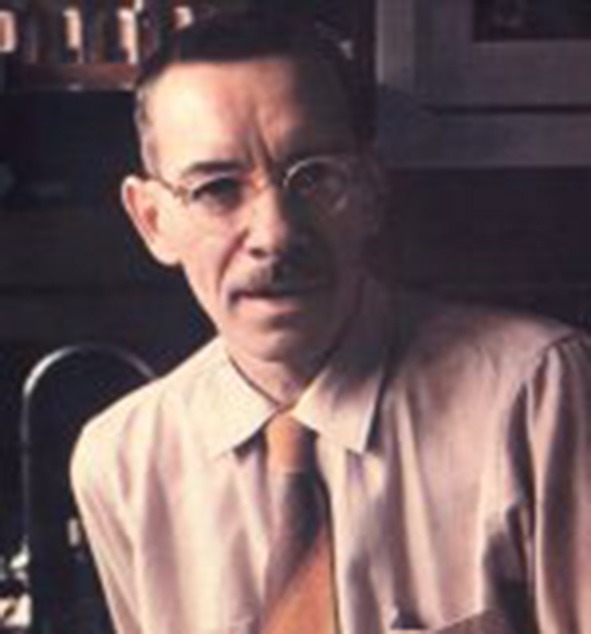
**Alfred Hershey (1908–1997)**. Source http://scarc.library.oregonstate.edu/coll/pauling/dna/people/hershey.html.

**Figure 2:**
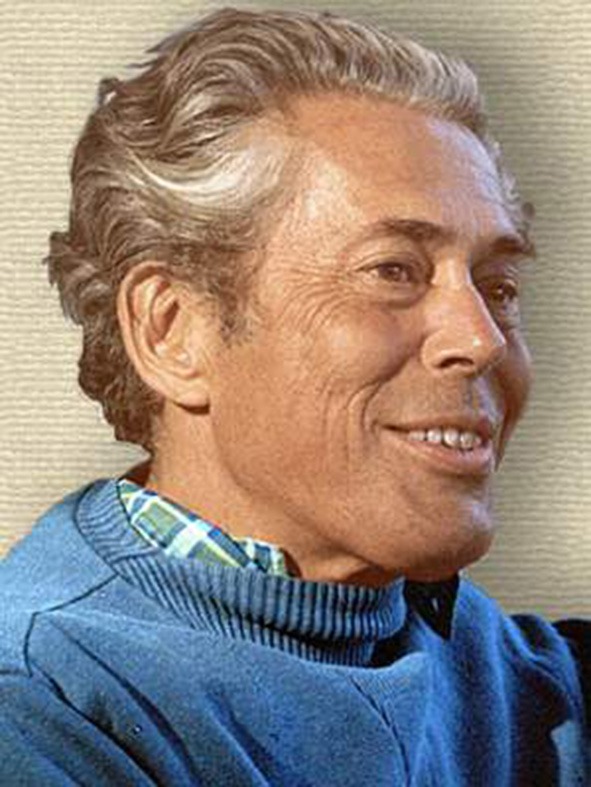
**Jacques Monod (1910–1976)**. Source: http://todayinsci.com/2/2_09.htm#MonodJacques.

The fog began to lift with the work of, among others, two people who later went on to become fathers of molecular biology, Alfred Hershey in the late 1930's and Jacques Monod in the 1940's (Figures 1, 2). Hershey (Hershey, 1939) (collaborating with his chairman, Jacques Bronfenbrenner) countenanced the use of a culture in the log phase of growth as the inoculum to start a new culture, thus dispelling the inviolable sanctity of the growth curve. Monod (1942) consigned the growth response of whole cultures to enzyme kinetics and showed that the rate of growth was dependent, in Michaelis–Menten fashion, on substrate concentration, while the yield was proportional to the amount of substrate available. These experiments were carried out with cultures growing in a steady state, a key point that I will return to shortly. Monod, probably dissatisfied by the prevailing view of the field as being superficial, soon looked elsewhere in his quest for molecular mechanisms. It is noteworthy that his studies on the regulation of gene expression originated from his growth physiological work on “diauxic growth,” a phenomenon wherein having glucose in the medium impedes the growth on other sugars. He left behind an encompassing yet dismissive parting shot (Monod, 1949): “The study of the growth of bacterial cultures does not constitute a specialized subject or branch of research: it is the basic method of microbiology.” As a discipline, physiology of bacterial growth came close to passing from confusion to oblivion in a single leap.

As is sometimes the case, subsequent work was facilitated by a clear definition. In Campbell (1957) proposed that the steady state growth condition be referred to as “balanced growth.” In so doing, he elevated what was previously just one phase in the growth curve (the log phase) into a general concept. In a sense, moving from the observation of log phase to the concept of balanced growth is like going from watching apples fall to thinking of gravity. Cells in balanced growth attain the maximum growth rate possible for that particular medium. One may fantasize a bacterium's most cherished ambition is to grow as fast as possible, thereby outpacing less productive competitors. But balanced growth has another important and unique attribute: it is the only *readily reproducible* growth condition. Consider how variable over time all the other states in the growth of a culture are. Sample now and sample a few minutes later, and you find that the cells already have different properties. Alas, even now this simple point is not always taken into consideration when defining research protocols. See a reasoned excoriation aimed at the practitioners of sloppy culturing by Neidhardt (2006). An untold amount of work carried out with cultures at undefined stages of growth is not reproducible, thus it is wasted.

The importance of growth at a steady state had been realized earlier, but Campbell's novel and precise term helped remove the aura of immutability from the growth curve. It provided the freedom to manipulate cultures by, for instance, repeatedly diluting them so as to maintain them in balanced growth. One of the most interesting of these manipulations in the early 1950s was the development of continuous cultures in chemostats (Monod, 1949; Novick and Szilard, 1950).

**Figure 3:**
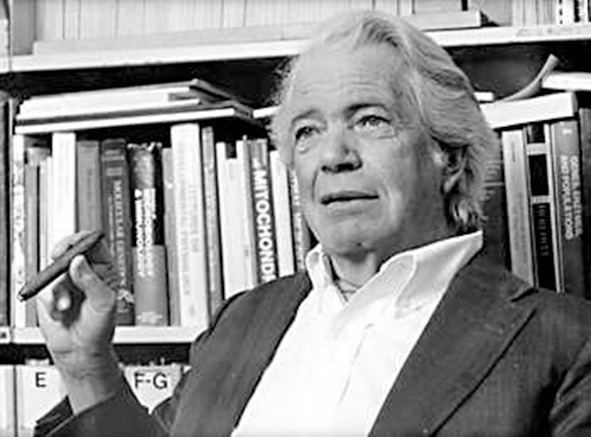
**Ole Maaløe (1914–1988)**. Source: http://www.denstoredanske.dk/Krop,_psyke_og_sundhed/Sundhedsvidenskab/L%C3%A6ger/Ole_Maal%C3%B8e.

Come the mid-1950s, growth physiology was extended to one of the main concerns of the day: the relationship of nucleic acids to protein synthesis. Here this narrative changes to a rather selective, personal account. It was in 1956 that I joined the lab of Ole Maaløe in Copenhagen (Figure 3). Eventually much work on growth physiology was to come from his lab and the people who had been there became known collectively as the “Copenhagen School” (Maaløe and Kjeldgaard, 1966; Cooper, 2008). The earliest finding, on which subsequent work relied, was that cells of one species growing at different rates (in balanced growth, of course!) differed in size depending on the growth rate, with the faster ones growing being larger. Consequently, cells growing in two different media but at the same growth rate have the same cell size. The Copenhagen lab was not alone in such studies (Schaechter et al., 1958). Extensive experiments relating RNA content to growth rate were also reported by Neidhardt and Magasanik (1960), Neidhardt (1963) and Herbert (1961). Thus, a sizable window was opened to molecular mechanisms and Monod was proven wrong to some degree.

I should mention that this work was made possible, as much as anything, by the rigor that Maaløe brought to experimental measurements. In his lab, viable counts were carried out so precisely that the experimental error was consistently smaller than random sampling error (and that was before accurate pipetting gadgets). Or, to determine the growth rate of a culture, optical density (mass) measurements were made *at least 10 times* in the course of each doubling of the culture. But the deeper point was a striving for a quantitative approach to studying growth.

Why would bacterial cells of the same species differ in size? Bacteria by dry weight consist mainly of proteins, so could fast growing cells be larger because they contain more protein-synthesizing ribosomes? When we measured the content of ribosomes in cells growing at different rates, we found, to our delight, that there was also a simple relationship here: the faster the growth rate, the more ribosomes per cell mass (Ecker and Schaechter, 1963). In other words, the concentration of ribosomes turned out to be a linear function of the growth rate. As if to test the rule, this relationship breaks down at very slow rates. This makes sense because otherwise cells growing infinitely slowly would have no ribosomes and would not be able to make proteins when placed in a richer medium. Eventually, the concentration of many other cellular components as a function of the growth rate became known in some detail (Bremer and Dennis, 1996). Because of such a dependency, bacteria obey the maxim of the Spanish philosopher José Ortega y Gasset that I am fond of quoting: “I am I and my circumstance” (*Yo soy yo y mi circunstancia*).

These studies deal with bacterial populations. How about single cells? Their life span is described by their cell cycle and is distinct from the growth curve. It depends instead on what happens between one division and the next. What events transpire during the cell cycle? It was observed early on using fairly simple microscopy and confirmed more recently by more sophisticated tools that the increase in mass in growing bacteria is exponential. In other words, growth is due to an autocatalytic expansion of most cell components. Constituents such as ribosomes and proteins are usually present in a large number of copies; therefore they need not all initiate their synthesis at once. One ribosome can be made now, another one an instant later, and yet their population will, in the aggregate expand exponentially. But the situation differs for elements that are present in one or a small number of copies, to wit, the chromosome and the cell itself. Being unitary events, both of these processes have to be regulated quite precisely, lest the population of cells become errantly heterogeneous. But in the 1950s and early 1960s, there were few tools with which to study the timing of DNA replication in single cells. Division synchronization of a culture could not readily be achieved without disturbing normal growth, e.g., by subjecting the culture to temperature shifts.

The earliest model for the regulation of the chromosome cycle was proposed by Helmstetter et al. (1968), Cooper and Helmstetter (1968) based on an expressly non-intrusive method to synchronize bacterial cells. They made use of the “baby machine,” a device to unobtrusively collect “newborn” cells. It was known that the *Escherichia coli* chromosome is composed of a single DNA molecule and that its replication starts at one site, the origin, and ends at another, the terminus. The H&C model proposed that the interval between initiation and termination is nearly constant at a given temperature, regardless of the growth rate and the richness of the medium. Regulation, therefore, is principally concerned with initiation, and this became the focus of such studies. But in fast growing cells, the time required for chromosome replication can be longer than the cell cycle. This led to the proposal that initiation need not wait for termination but can take place before the previous replication finishes, thus leading to multiple concurrent replication events on a chromosome—the so-called “multifork replication” (Yoshikawa and Sueoka, 1963).

These ways of thinking led to subsequent investigations into the mechanisms that control bacterial gene expression and chromosome replication. How is the synthesis of the ribosomal RNAs and proteins regulated? What might this have to do with the control of gene expression? How is chromosome replication regulated? And so on. I have participated in this work and derive much pleasure from the sophisticated understanding of the mechanisms that have been unraveled. However, I still stand in awe of the central marvel—the ability of such seemingly simple cells to grow in such perfect rhythm. For a lucid manifesto of this outlook, see the commentary by Neidhardt (1999).

Studies on the mechanisms that regulate growth were greatly aided by genetic analysis. A large number of conditional mutants, especially of *E. coli*, were constructed, e.g., some heat sensitive (see Hirota et al., 1968), some cold sensitive (see Ingraham, 1969). Studying their phenotype at the restrictive temperatures revealed much about the biochemical basis for growth and became an essential complement to the purely physiological experiments.

## The present day

Although the Copenhagen School emphasized a quantitative approach, early on at least, the mechanistic understanding of growth phenomena was undeniably limited. Francis Crick figured that out that appallingly fast. When I visited him at the Cavendish Laboratory in Cambridge University in 1958, he blurted out: “Congratulations! You people started a new field, but it's over!” Gulp! In a narrow sense, this was true for the time, although even then I could have timidly argued that the physiological focus on the growing cell had contributed a needed counterpoint to molecular reductionism. But it took time. For some 50 years, until around the turn of the 20th century, growth physiology remained more or less in a latent state.

Recently, microbial growth physiology has seen a rebirth in a form that seeks a deeper *quantitative* understanding of phenomena on a whole cell level. This is exemplified by the emergence of systems biology: an approach made possible by technologies that can gather and analyze colossal amounts of information to disclose how intracellular transactions are interrelated. In fact, I have heard it said that systems biology is just an all-embracing view of cell physiology, or, if you wish, a continuation of the escape from biochemical reductionism. As has been true throughout history, research into microbial physiology continues to be guided by the development of new methods of experimental and mathematical analysis. A few examples (of many) can be seen in the exciting papers by Edwards et al. (2001), Wang et al. (2010), Valgepea et al. (2013), Klumpp and Hwa (2014), and Scott et al. (2014).

How is the bacterial growth physiology of old connected to the systems biology of today? Both historical and conceptual threads are clearly visible (Schaechter, 2006). Old questions, such as how many macromolecular components are in a cell, how rapidly are they made, and how do their interactions result in cell growth, can now be studied with modern tools. Yet, the newer methods still have a direct connection with the older ones. An example is the proteomic measurement of growing versus stressed *E. coli*, first done on a large scale in Neidhardt's lab (2011). The initial impetus for this work was to determine the number of proteins made at different growth rates of the culture, which was soon directed to looking at the effects of physiological stresses. But this approach was quickly replaced when these researchers realized that such studies had been focused largely on what *the investigator* thought interesting, useful, or potentially vital to the cell. Soon, they saw that the new methods of surveying the global production of proteins, notably two-dimensional gel electrophoresis, enabled the investigator to put the ball in the microbe's court and discover what *the cell* deemed important. Many such studies of the proteins made at different growth rates and temperatures, as well as when under various stresses, led to a nuanced appreciation of the cell as a dynamic system, with an expanded universe of rules and relationships governing its physiology and metabolism.

A major value of systems biology lies in its ability to create predictive models, something that has been achieved to a considerable extent with yeast and is being realized with bacteria. We are beginning to get a multidimensional view of the complex network of interactions that leads to the growth of a cell. As ever, the experimental basis for this work must be growing the cells under reproducible and readily assayable conditions, in other words, using cultures in balanced growth as the baseline condition. This is but one of the concepts that systems biology inherits from growth physiology.

*Enfin*, aficionados of balanced growth, such as myself, are often reminded that this state is unusual in nature. This is not the fault of the cells. Most planktonic cells and possibly many sessile ones grow as rapidly as conditions permit (although the abundant cyanobacteria in the ocean respond to non-nutritional inducements, such as their diel clock). Microbial environments are highly variable and usually allow only short spurts of unhindered growth that follow the infusion of foodstuff. Balanced growth over protracted periods is found mainly in the laboratory. But the experimenter who provides conditions that permit balanced growth is doing no more than letting cells put into action their fundamental yearning to grow. The cells take care of everything else.

### Conflict of interest statement

The author declares that the research was conducted in the absence of any commercial or financial relationships that could be construed as a potential conflict of interest.
